# Alloparenting is associated with reduced maternal lactation effort and faster weaning in wild chimpanzees

**DOI:** 10.1098/rsos.160577

**Published:** 2016-11-09

**Authors:** Iulia Bădescu, David P. Watts, M. Anne Katzenberg, Daniel W. Sellen

**Affiliations:** 1 Department of Anthropology, University of Toronto, Toronto, Ontario, CanadaM5S 2S2; 2 Department of Anthropology, Yale University, New Haven, CT 06511, USA; 3 Department of Anthropology and Archaeology, University of Calgary, Calgary, Alberta, CanadaT2N 1N4

**Keywords:** alloparenting, allocare, lactation, stable isotopes, weaning, nursing

## Abstract

Alloparenting, when individuals other than the mother assist with infant care, can vary between and within populations and has potential fitness costs and benefits for individuals involved. We investigated the effects of alloparenting on the speed with which infants were weaned, a potential component of maternal fitness because of how it can affect inter-birth intervals, in wild chimpanzees (*Pan troglodytes schweinfurthii*) at Ngogo, Uganda. We also provide, to our knowledge, the first description of alloparenting in this population and present a novel measure of the contribution of milk to infant diets through faecal stable nitrogen isotopes (δ^15^N). Using 42 mother–infant pairs, we tested associations of two alloparenting dimensions, natal attraction (interest in infants) and infant handling (holding, carrying), to the proportion of time mothers spent feeding and to maternal lactation effort (mean nursing rates and mother–infant δ^15^N differences). Neither natal attraction nor infant handling was significantly associated with feeding time. Infant handling was inversely associated with both measures of lactation effort, although natal attraction showed no association. Alloparenting may benefit mothers by enabling females to invest in their next offspring sooner through accelerated weaning. Our findings emphasize the significance of alloparenting as a flexible component of female reproductive strategies in some species.

## 1. Introduction

Alloparenting, when individuals other than the mother assist with infant care, occurs to varying degrees in birds and in many mammals, including rodents, social carnivores, cetaceans and primates [1–5]. The nature of interactions and the age/sex classes of the individuals involved are well documented in several taxa, and these reports have been used to infer the adaptive benefits, but also the potential costs, of alloparenting to the individuals involved [1,3,6–10]. For mammalian mothers, benefits include the promotion of earlier weaning of their infants, which leads to shorter inter-birth intervals and increased reproductive success, so long as earlier weaning does not compromise infant survival [1,3,5,6,8,11–13]. This is because frequent alloparenting may give mothers more opportunities to feed freely without their infants, which may increase their net energy gains and allow them to invest more energy in milk synthesis [6,14,15]. It may also lead to increased milk nutrient density and energy content, thus to greater nutrient transfer to offspring, faster infant development and earlier ages at which infants are successfully weaned [14,16–19]. In addition, frequent alloparenting may lead to quicker weaning because infants who receive more alloparenting experience longer periods between nursing bouts than infants who have more constant access to their mothers' nipples, which can compromise lactation and lead to a quicker resumption of cycling for mothers [11,20]. Species in which alloparenting is common have infants that grow faster and are weaned earlier relative to body size than related species in which alloparenting is less common or absent [1,7,9,10,21]. In cooperatively breeding mammals, in particular, alloparents can improve the fitness of breeders by helping parents to meet the energy needs of raising infants, either directly (e.g. by provisioning mothers, infants or both and/or by carrying infants) or indirectly (e.g. by allowing mothers to spend more time feeding and to feed more efficiently, and by reducing the amount of energy mothers need for transporting infants). Both types of effects can enhance infant size and growth rates because the total amount of energy available for growth is more than mothers could provide if they were responsible for all infant care (e.g. common dwarf mongoose, *Helogale parvula* [7]; prairie voles, *Microtus ochrogaster* [22], marmosets and tamarins, *Callitrichidae* spp. [14,15]; humans, *Homo sapiens* [23]; meerkats, *Suricata suricatta* [24], banded mongoose, *Mungos mungo* [25]). Alloparenting can also differentially affect the fitness of mothers among and within populations of the same species because the rates and types of interactions are not always consistent across individuals (e.g. meerkats, *S. suricatta* [26]; sperm whales, *Physeter macrocephalus* [4]; black-and-white ruffed lemurs, *Varecia variegata* [27]; humans, *H. sapiens* [28]). In some cases, alloparenting may seemingly have no direct fitness benefits to mothers (e.g. European badgers, *Meles* [29]; degus, *Octodon degus* [30]), but this behaviour can still be beneficial to alloparents through a variety of mechanisms [8].

The potential costs associated with alloparenting include expending time and energy on caring for others' offspring with no apparent direct benefits to alloparents, and less competent, rough or excessive care that can lead to negative health effects for infants and be stressful for mothers [10,12].

Studies investigating the effects of alloparenting on maternal fitness in species that are not cooperative breeders are concentrated on a handful of non-human primates (i.e. vervets, *Chlorocebus pygerythrus* [11]; siamangs, *Symphalangus syndactylus* [31,32]; black-and-white ruffed lemurs, *V. variegata* [27]). While alloparenting frequencies, types of interactions and the individuals involved are well documented in many primate taxa, the adaptive value of alloparenting remains understudied and unresolved [8,33].

Chimpanzees (*Pan troglodytes*) are among those primates in which alloparenting occurs, but is neither obligatory (chimpanzees are not cooperative breeders) nor notably common [5]. Nevertheless, its occurrence raises questions about potential costs and benefits to mothers, infants and alloparents. We examined the potential adaptive value of alloparenting for mothers in one large community of wild eastern chimpanzees (*Pan troglodytes schweinfurthii*) using data on 42 mother–infant pairs and a novel measure of the contribution of maternal milk to infant diets obtained through analysis of faecal stable isotopes. With approximately 55 adult females and a total of around 200 individuals, Ngogo, in Kibale National Park, Uganda is the largest chimpanzee community currently being studied [34]. High food abundance at the site helps to explain the large community size [34] and results in low female feeding competition; both of these factors help to explain why many natal females at Ngogo reproduce in the group, despite the tendency for female dispersal in chimpanzees [35–37]. This is, to our knowledge, the first study to describe alloparenting in chimpanzees at Ngogo, where interactions with individuals other than the mother occur frequently for some infants but are less frequent for others (see Results). Investigating the effects of alloparenting on lactation and weaning in a wild ape population can help reveal the adaptive value of this behaviour in social mammals other than the well-studied cooperative breeders, and can shed light on the evolutionary trajectory that led to strategies of shared infant care in humans [5,23].

### 1.1. Maternal lactation effort and weaning

Directly examining the effects of alloparenting on female reproductive success in most primates is difficult because their long lifespans, late weaning ages, long inter-birth intervals and small number of surviving offspring mean that obtaining sufficiently large samples requires long-term studies [21]. In cross-sectional investigations, infant nursing rates can be used to measure the speed with which infants are weaned because they are positively correlated with milk synthesis and are thus a proxy measure for maternal lactation effort [17,38,39]. Infants who nurse less often will probably have mothers that stop lactating and resume ovulating sooner [16,17,38,39]. However, nursing rates may not be a good indication of milk intake, because infants who nurse less often could obtain more milk in each nursing bout than infants who nurse more often [17,38,39]. Milk nutrient density can also vary across mothers and at different stages of lactation, and to meet nutritional demands of growth and development, some infants may need greater volumes of milk when it is more dilute, but lower volumes when it more nutrient rich [39–43]. Moreover, nipple contact and even suckling does not guarantee that infants are ingesting milk, because infants sometimes make nipple contact for comfort only (e.g. rhesus macaques, *Macaca mulatta* [44]; Hanuman langurs, *Presbytis entellus* [45]; eastern chimpanzees, *P. t. schweinfurthii* [46]).

A novel method that uses faecal stable nitrogen isotopes (δ^15^N) overcomes some of the limits on observational data by measuring the contribution of maternal milk to infant diets directly. Data on δ^15^N can be used along with nursing observations to assess inter-individual variation in the rate, or speed, with which weaning occurs [46,47]. Stable nitrogen isotopes in hair, dentine, bone collagen and blood serum have been used to investigate lactation effort, weaning and maternal care strategies in several mammals (e.g. northern fur seals, *Callorhinus ursinus*; California sea lions, *Zalophus californianus* [48]; beluga whales, *Delphinapterus leucas* [49]; Steller sea lions, *Eumetopias jubatus* [50]; rhesus macaques, *M. mulatta* [51]; eastern chimpanzees, *P. t. schweinfurthii* [52]; meerkats, *S. suricatta* [53]; cave bears, *Ursus spelaeus* [54]). Stable nitrogen isotopes in faeces can be obtained non-invasively and show day-to-day changes in milk intake, because δ^15^N ratios reflect nitrogen derived primarily from the amino acids of dietary proteins [47,55–57]. Like enrichment in collagen or keratin, the faeces of exclusively suckling primate infants exhibit δ^15^N values that are 2–3‰ (permil) higher than the faeces of their mothers [47]. In another paper [46], we showed that faecal stable nitrogen isotopes provide a physiologically meaningful way of documenting infant nutritional development in chimpanzees and can reliably determine the timing of feeding transitions in this population. Infant chimpanzees at Ngogo showed a maximum 2‰ elevation in faecal δ^15^N above that of the mother when they were less than or equal to 1 year old [46]. A subsequent steady decline in δ^15^N values with increasing age indicated a gradual weaning process that lasts over several years [46,52,58]. A gradual weaning process also occurs in contemporary foraging human populations [59], and has been documented using stable isotopes from bone collagen in past human groups [60] and from dentine in chimpanzees at Taï, Côte d'Ivoire [52]. Faecal stable nitrogen isotopes at Ngogo showed that, on average, the end of the weaning process occurred when offspring were 4–4.5 years old, which fits with the average of 4–5 estimated from behavioural observations of weaning at other chimpanzee sites [61,62]. Inter-individual variation in weaning age occurs at Ngogo [46], as in other mammals (e.g. primates: mountain gorillas, *Gorilla beringei beringei* [63]; humans, *H. sapiens* [64,65]; rhesus macaques, *M. mulatta* [51]; eastern chimpanzees, *P. t. schweinfurthii* [52]; non-primates: northern fur seals, *Ca. ursinus*; California sea lions, *Z. californianus* [48]; beluga whales, *D. leucas* [49]; Steller sea lions, *E. jubatus* [50]).

### 1.2. Alloparenting in chimpanzees

Detailed descriptions of alloparenting in wild chimpanzees are lacking and documented cases to date are based on small sample sizes [66–69], partial measures that included a few behaviours (e.g. playing [70]), or in specific, but unusual, contexts (e.g. adoption [71]). The shortage of detailed investigations may be because alloparenting is uncommon in most chimpanzee populations, perhaps because of maternal protectiveness given threats of predation or infanticide [72–74]. In addition, chimpanzee females usually disperse from their natal groups to live among unrelated individuals, and while alloparenting by non-kin occurs [3,12,67], in the absence of supportive kin, primate mothers are typically protective of their young infants and restrict the ability of others to interact with them [5,8,10]. Indeed, reports of alloparenting in chimpanzees typically involve mothers permitting older siblings or other maternal kin to hold and carry infants and alloparenting by non-kin is relatively less common [5,68,69]. At Mahale in Tanzania, for instance, mothers and infants were eight times more likely to resist alloparenting by nulliparous female non-kin than kin, and non-kin nullipara were twice as likely to groom mothers before they could interact with infants [68].

Chimpanzee societies are characterized by high fission–fusion dynamics: community members associate in temporary subgroups that vary in size, duration and composition and are sometimes alone, or, in the case of females, accompanied only by dependent offspring. Thus mothers have options about the extent to which they associate with individuals other than their own dependent offspring and with which others they associate. Moreover, female gregariousness varies widely at Ngogo [35,75]; combined with variation in maternal parity and in whether mothers have non-independent juvenile offspring and/or associate with adult daughters who have not dispersed, this means that females with unweaned infants face wide variation in opportunities to receive alloparental care. Female dominance hierarchies are absent at Ngogo, and while conflicts between females do occur, decided outcomes are rare [35].

### 1.3. Dimensions of alloparenting: natal attraction and infant handling

We focused on two separate suites of behaviours associated with alloparenting in chimpanzees; natal attraction and infant handling, which are important to distinguish because their fitness costs and benefits can differ [5,8,33]. Individuals other than mothers can show interest in infants through touching, peering, playing or grooming (natal attraction), or they may hold or carry infants (infant handling) [76–79]. Natal attraction in primates often occurs when infants are in body contact with their mothers, which means that infants may continue to have maternal nursing access, and reveals the individuals most interested in interacting with infants. Infant handling occurs when infants are not in physical contact with their mothers, which means that maternal nursing access is hindered, and depends more on the extent to which mothers tolerate the interactions of others with their infants [5,8,33,79,80].

### 1.4. Hypotheses and predictions

We assessed whether chimpanzee mothers benefit from alloparenting because it promotes quicker weaning, presumably by positively influencing energy balance. We compared rates of natal attraction and infant handling to one proxy for maternal energy intake, the proportion of time mothers spent feeding, and to two measures of lactation effort (mean nursing rates and mother–infant δ^15^N differences). If mothers benefit from alloparenting, mothers of infants who were handled more should spend more of their time feeding because they would have more opportunities to forage away from their infants [6,12,14]. Increased feeding time could lead to higher net energy gains and higher rates at which females transfer nutrients to their infants. We recognize that feeding time is not necessarily an accurate assay of energy intake because of variation in processing time and energy content among foods. Thus we see feeding time data as providing an initial test and we hope to collect more accurate data on energy balance in the future. Additionally, however, mothers whose infants receive much handling could have relatively low lactation effort because they have relatively long intervals between nursing bouts. Increased energy gains and longer inter-bout intervals should accelerate weaning [6,11,14,20] and should be evident in relatively low mean δ^15^N differences. We expected that natal attraction would not affect the time mothers spent feeding or their lactation effort because primate infants are usually in body contact with their mothers when receiving natal attraction [33,79,80]. Mothers would, therefore, not be afforded extra opportunities to feed freely, and their infants could continue to obtain milk on demand.

Failure to find a positive relationship between the amount of alloparental care received and maternal feeding time would be inconsistent with the hypothesis that mothers benefit from the care, although with the caveat that feeding time does not necessarily measure the rate of energy intake accurately. Failure to find a significant relationship between allocare and lactation effort, however, would be incompatible with the accelerated weaning hypothesis, although it would not rule out the possibility that they can benefit from increased infant survival.

## 2. Material and methods

### 2.1. Study site and species

Ngogo is in Kibale National Park, Uganda. The 35 km^2^ study area comprises mostly dry-ground forest at various successional stages, including largely old growth adjacent to colonizing forests that were once grasslands, plus areas of swamp forest, bush and anthropogenic grasslands [34,81]. The Ngogo chimpanzee community is the largest ever recorded; during this study it included between 202 and 207 individuals (54–57 adult females, 31–33 adult males, 30–32 immature females, 33 immature males, 22–28 infant females, 22–25 infant males). The chimpanzees have been under constant observation since 1995 and are well habituated to the presence of researchers as they have been studied since the early 1990s [34].

### 2.2. Study subjects

Our study subjects were 42 mothers and their infants who varied in age from 1 day old to 6 years old at the start of data collection. We assigned infants to age categories, defined as 0 to less than or equal to 1, 1 to less than or equal to 2, 2 to less than or equal to 3, 3 to less than or equal to 4, 4 to less than or equal to 5, 5 to less than or equal to 6 and 6 to less than or equal to 7. Eighteen study subjects contributed data to multiple age categories because they grew older during data collection; thus the total sample size was 62 infants by age category (table 1). All infants made regular nipple contacts with their mothers and thus had measurable nursing rates at each of the age categories included in the analyses.

**Table 1.** Study subjects, focal sampling hours and instantaneous scans.

infant age category (years old)	number of infants	infant mean number of focal hours (s.d.)	mother mean number of scans (s.d.)
0 to ≤1	12	17.6 (7.3)	210 (85)
1 to ≤2	13	11.9 (5.4)	142 (63)
2 to ≤3	16	11.4 (5.6)	134 (65)
3 to ≤4	14	12.2 (5.6)	142 (62)
4 to ≤5	3	14.3 (3.0)	167 (38)
5 to ≤6	3	9.8 (4.5)	115 (47)
6 to ≤7	1	39.2 (0.0)	244 (0)
total	62^a^	13.4 (6.9)	155 (71)

^a^Sixty-two infants by age category from 42 different individuals (see Material and methods).

### 2.3. Behavioural data collection and analyses

From January to March 2013 and September 2013 to June 2014, I.B. used focal sampling [82,83] to collect continuous data on natal attraction, infant handling and nursing. Samples lasted 1 h and were terminated if the focal infant was out of view for more than 10 min. Data included all behavioural acts directed to, and received by, infants. We did not include time out of view when calculating the total number of focal hours in our analyses. To maximize independence between samples, those on a given infant were separated by at least 30 min unless the infant changed its behavioural state; for instance, if an infant changed from ‘resting’ to ‘feeding’ before the 30 min had passed, we could start a new focal sample on that infant [76]. Otherwise, at the end of a sample, I.B. switched to another infant if one was visible, and subsequently tried to cycle through data collection on all infants present, in the same order, for the rest of the day. Selection of focal infants was often random but if there was an infant available on which data were lacking (for instance, because it was a newborn or the mother–infant pair was generally more difficult to locate), we specifically tried to sample them.

Nursing bouts were defined as infants making nipple contact. This did not include time infants spent on their mothers' ventrums with their faces not visible (which made it unclear whether nursing was occurring), and the duration of this time was subtracted from the total number of focal hours for each infant. Infant handling occurred when individuals other than the mother held or carried infants [76]. Natal attraction included intense peering at infants; touching, grooming or playing with them; and attempting to transfer (e.g. pulling on infants) or actually transferring them from their mothers [76]. No allonursing or attempts to allonurse were seen. Interactions between infant peers were not included as natal attraction or infant handling. We counted both natal attraction and infant handling when these occurred in sequence, but if an individual simultaneously engaged in both kinds of behaviours (e.g. held an infant while simultaneously grooming or peering at it), we counted this as a bout of infant handling only [76]. Occasionally, an infant was near but not in body contact with the mother, and an alloparent groomed or played with the infant without holding it. This was considered natal attraction [76] because the infant could still move with ease between interacting with the alloparent and returning to the mother to nurse and because it contrasts with actual holding, when the alloparent can potentially handle the infant clumsily or roughly and risks dropping it. Occasionally, an alloparent who was grooming or playing with an infant not in maternal body contact tried to pick up the infant and the infant's mother then quickly retrieved it; this suggests that chimpanzee mothers may consider holding of infants costlier than independent grooming or playing.

We obtained 831 focal sampling hours and a mean of 13.4 focal hours per infant by age category (±s.d.: 6.9, range: 4.4–39.2; table 1). We calculated hourly behavioural rates by dividing the number of bouts of natal attraction, infant handling or nursing for every infant by the total hours of focal sampling observations at each age category. Behaviours were considered distinct bouts when they were separated by at least 1 min [16,84,85]. We did not include durations of natal attraction or infant handling in analysis here because these can vary from a few seconds to more than an hour and we were often not able to obtain complete durations of bouts that had either started before the focal sample began or continued past the end of the focal sample.

During the focal animal samples, I.B. also conducted 5 min instantaneous scans to record the state behaviour of the focal infant's mother [83]. Mothers were feeding if they were ingesting food, chewing ‘wadges’ or looking for food items. Other behavioural states included resting, behaviours directed to own infant (e.g. nursing, grooming, playing), socializing with individuals other than own infant (e.g. grooming, copulating), travelling and self-directed behaviours (e.g. autogrooming). We excluded scans for which we were unsure of the mother's behaviour. We obtained 9579 instantaneous scans and a mean of 155 instantaneous scans of mothers per infant by age category (±s.d.: 71, range: 52–325; table 1). To calculate the proportions of time that a mother spent feeding at each of her infant's age categories, we divided the number of instantaneous scans during which she was feeding by her total number of scans for that category.

We included three covariates in the analyses because of their potential effects on maternal and alloparental behaviours. First, we included maternal parity as a covariate because of its possible influence on milk quality, lactation performance and mothering experience [39,41,86]. Maternal parities were known from long-term demographic records of the Ngogo chimpanzee population. Mothers were either primiparous (first-time mothers) or multiparous (have had more than one infant). Second, we included the sex of infants as a covariate because this can affect the lactation performance of mothers [41,42] and the amount of alloparenting infants receive [76], and because sex differences in rates of weaning and development are known to occur in primates, including chimpanzees [52,87]. The sex of study infants was determined from observations of their genitalia. Third, we included infant age to account for differences in nursing and milk consumption at different developmental stages [46,52]. Infant age estimates were based on the appearance of infants (and their mothers) when observers first saw them, and age at first sighting varied from 1 day to several months. We assigned infants to yearly age categories for purposes of analysis.

### 2.4. Faecal sample collection and stable isotope analyses

Between September 2013 and June 2014, I.B. and five trained field assistants collected faecal samples from infants and their mothers. Faecal samples were desiccated on site using a solar food dehydrator and frozen until transported to the University of Calgary's Isotope Sciences Laboratory for processing and laboratory analyses. The reader is referred to Bădescu *et al*. [46] for detailed descriptions of the stable isotope protocols we followed. Isotopic compositions of different foods vary, and thus, day-to-day variation in the faecal stable isotope values from the same individual are common [46,88]. Therefore, each faecal sample collected from an infant in this study was matched by a sample collected from the mother on the same day (matched samples). Using matched samples allowed us to control for differences in the isotopic compositions of different foods and to hone in on dietary differences owing to nursing because once infants start to ingest solid food, mothers and dependent offspring feed together on the same vegetation in the same parts of the canopy; thus any day-to-day differences in faecal stable isotope values between mothers and infants should be owing to maternal milk ingested by infants [46,47]. We calculated differences in faecal stable nitrogen isotopes (δ^15^N‰) of matched samples and obtained a mean of all matched sample differences for that infant by its age category. We only used δ^15^N values of infants for which we had obtained at least three matched samples. We included mean δ^15^N differences of matched samples for 34 out of the 62 infants by age category.

### 2.5. Statistical analyses

We used two generalized estimating equations (GEEs) analyses to assess how rates of (i) natal attraction and (ii) infant handling correlated with nursing rates, mean faecal δ^15^N differences between infants and mothers, proportion of time mothers fed and the covariates maternal parity, infant age and infant sex. We included infant identities as random effects to control for repeated measurements of the same subjects. We used GEE analysis instead of generalized linear models or generalized linear mixed models analyses because GEEs permit dependent variables to be correlated, allow repeated measures of the same individuals, work well with non-standard data (e.g. binary and count variables) and can handle some missing values such as missing δ^15^N differences [89–91]. To confirm that the reliability of GEEs was not affected by the missing δ^15^N values, we conducted separate GEEs only including those infants by age category (*n* = 34) for whom we had mean δ^15^N differences. The results were nearly identical to the GEEs that included the infants with missing δ^15^N values, and significance or non-significance remained unchanged. We, therefore, present results of the GEEs including all study infants. When performing GEE analyses, we considered changes in the quasi-likelihood ratios (i.e. goodness of fit ratios between the quasi-likelihood indicators; corrected quasi-likelihood under the independence model information criterion (QIC) and uncorrected QIC), which are in the ‘smallest is better’ form, and were monitored to ensure that adding numerous covariates did not decrease the precision of the output results. GEEs were run in SPSS (v. 23) with the identity link function and alpha set at *p* = 0.05.

## 3. Results

Out of the 42 different infants, 39 received some natal attraction and 25 were handled. Overall, natal attraction occurred at a rate of 0.60 bouts per hour (±s.d.: 0.60; range: 0.00–3.30) and infant handling at 0.12 bouts per hour (±s.d.: 0.19; range: 0.00–0.70). For those infants who received alloparenting, natal attraction occurred at a mean rate of 0.68 bouts per hour (±s.d.: 0.59; range: 0.04–3.30), and infant handling at 0.25 bouts per hour (±s.d.: 0.19; range: 0.04–0.70). The mean rate of nursing was 1.1 bouts per hour (±s.d.: 0.48; range: 0.15–2.52) and the mean δ^15^N difference was 0.50‰ (±s.d.: 0.54; range: −0.14–2.05). The mean proportion of time mothers spent feeding was 0.43 (43% of time; ±s.d.: 0.13; range: 0.15–0.82).

**Figure 1.**
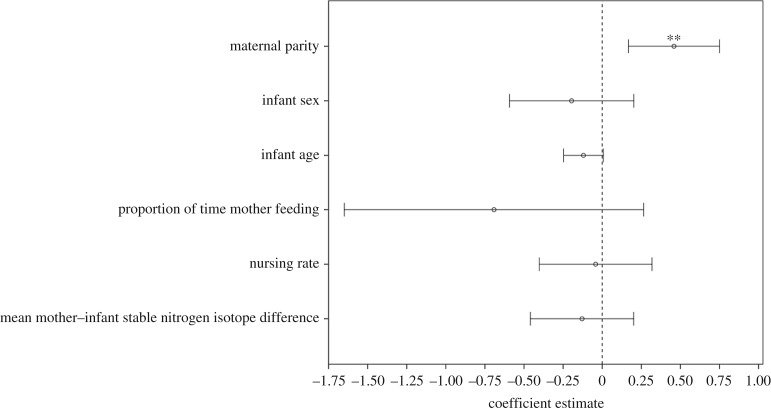
GEE *β*-coefficient estimates (circles) and their 95% confidence intervals (CIs; bars) for the effects of natal attraction on each dependent variable and covariate. Nursing rate, mean mother–infant δ^15^N difference and the proportion of time the mother was feeding were continuous variables, infant age was an ordinal variable, while maternal parity (primiparous, multiparous) and infant sex (female, male) were binary variables. **p < 0.01.

**Figure 2.**
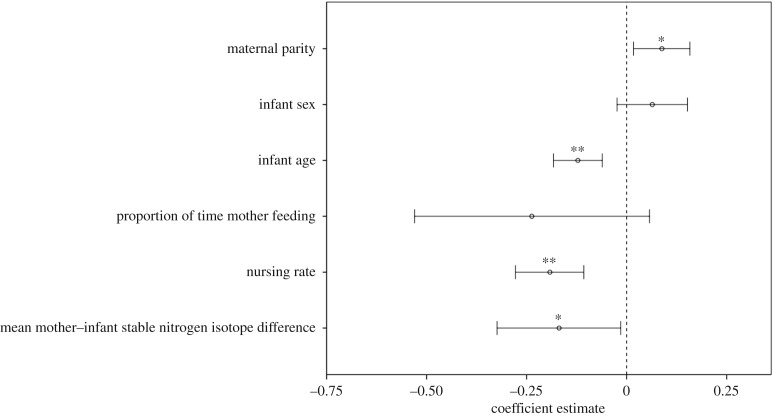
GEE *β*-coefficient estimates (circles) and their 95% CIs (bars) for the effects of infant handling on each dependent variable and covariate. Nursing rate, mean mother–infant δ^15^N difference and proportion of time the mother was feeding were continuous variables, infant age was an ordinal variable, while maternal parity (primiparous, multiparous) and infant sex (female, male) were binary variables. *p < 0.05; **p < 0.01.

Infants of multiparous mothers received more alloparenting than infants of primiparous mothers (attraction: *p* < 0.01, handling: *p* < 0.05; table 2). Neither natal attraction (*p* = 0.34) nor infant handling (*p* = 0.16) differed significantly according to infant sex. Younger infants received more alloparenting than older infants, and while the effect of infant age was only significant for infant handling (attraction: *p* = 0.06; handling: *p* < 0.001), the beta value (*β*), which indicates how strongly alloparenting correlated with infant age, was the same for both natal attraction and infant handling. Alloparenting did not affect the proportions of time mothers spent feeding (attraction: *p* = 0.16; handling: *p* = 0.11). Natal attraction did not affect nursing rates or mean δ^15^N differences (*p* = 0.50 and *p* = 0.34, respectively; figure 1). Higher rates of infant handling were associated with lower nursing rates (*p* < 0.001) and lower mean faecal δ^15^N differences between infants and mothers (*p* < 0.05; figure 2).

**Table 2.** GEE results for effects of alloparenting on covariates (maternal parity, infant age and sex), lactation effort (nursing, δ^15^N) and proportions of time the mother was feeding. (*p < 0.05, **p < 0.01.)

	dimensions of alloparenting							
	natal attraction				infant handling			
dependent variables and covariates	*β*	Wald's *χ*^2^ (d.f. = 1)	s.e.	*p-*level	*β*	Wald's *χ*^2^ (d.f. = 1)	s.e.	*p-*level
maternal parity	0.46	9.54	0.15	0.002**	0.09	5.89	0.04	0.02*
infant sex	−0.20	0.93	0.20	0.34	0.06	2.02	0.04	0.16
infant age	−0.12	3.43	0.06	0.06	−0.12	15.45	0.03	0.00001**
proportion of time the mother was feeding	−0.69	2.01	0.49	0.16	−0.24	2.50	0.15	0.11
nursing rate	−0.04	0.05	0.18	0.82	−0.19	19.42	0.04	0.00009**
mean mother–infant δ^15^N difference	−0.13	0.59	0.17	0.44	−0.17	4.60	0.08	0.03*

## 4. Discussion

Our results mostly support the hypothesis that alloparenting in the form of infant handling benefits mothers at Ngogo by accelerating weaning of their infants. Mothers of infants who were handled more had relatively low lactation effort: their infants nursed less often than infants of mothers who received less handling and showed smaller δ^15^N differences, which means that milk contributed less to their age-specific diets. Reduced maternal lactation effort leads to earlier weaning and enables females to invest in their next offspring sooner, which can lead to shorter inter-birth intervals and higher reproductive success so long as early weaning does not compromise infant survival [6,8,11,16,17,22,38,92]. Inter-birth intervals of female chimpanzees at Ngogo are relatively short in general (D. P. Watts *et al*. 1995–2016, unpublished Ngogo data), although we have not yet investigated the relationship between infant handling and interval length. Moreover, infant mortality in the first year is relatively high at Ngogo, but thereafter it is much lower than in other chimpanzee populations for which data exist, and mortality for weanlings (4–6 years) at Ngogo is zero so far [93], which implies that accelerated weaning does not have survival costs.

As expected, natal attraction was not associated with reduced maternal lactation effort, which is what is expected if these two suites of behaviours are under different selection pressures. This emphasizes the importance of separating natal attraction and infant handling when studying the potential fitness impacts of alloparenting, as they probably present different costs and benefits [76,79]. Excluding ‘kidnapping’, which has not been seen at Ngogo, infant handling is a good indication of which individuals mothers permit to hold their infants [8,94]. We can, therefore, expect that the costs and benefits associated with handling are mostly a result of maternal consent that the fitness impacts of infant handling mostly favour mothers [8]. By contrast, natal attraction can be stressful for mothers and may have negative fitness consequences when mother–infant dyads are harassed by individuals who are keen on handling but are not permitted to do so (e.g. yellow baboons, *Papio cynocephalus* [94]).

Contrary to our prediction, we did not find that alloparenting afforded mothers more time to feed. Studies of several non-primate mammals have yielded similar results (e.g. prairie voles, *Mi. ochrogaster* [22]; bottlenose dolphins, *Tursiops truncatus* [2]), but data from other primates in which the relationship between maternal time allocation and alloparenting has been investigated typically shows positive relationships between alloparenting and maternal feeding time (e.g. marmosets and tamarins, *Callitrichidae* [14]; siamangs, *Sy. syndactylus* [31,32]; black-and-white ruffed lemurs, *V. variegata* [27]). Even though we did not find that receipt of alloparenting was positively correlated with the amount of time that mothers spent feeding, those mothers whose infants were handled more could still have benefited because they could forage more efficiently and thereby increase their net energy gain when they were not encumbered by infants and/or because they expend less energy on carrying young [6,10,12,15,31,32].

The mechanism by which alloparenting at Ngogo reduced maternal lactation effort and accelerated weaning may not have been through improved net energetic gains. Instead (or in addition), mothers whose infants were subjected to relatively frequent handling might have had longer intervals between nursing bouts [11,20]. A similar negative correlation between alloparenting and lactation effort was found in a human study of 18 827 children and their mothers, as frequent alloparenting was associated with lower levels of breastfeeding [28]. The duration of lactation is influenced by mechanical stimulation of the nipple that leads to the release of the hormone prolactin, which inhibits ovulation. Since prolactin levels increase upon initial suckling stimulus and remain elevated for up to two hours after the end of a suckling session [85], higher nursing rates promote lactation and suppress ovulation [38,95]. Indeed, studies on infant nursing in rhesus macaques (*M. mulatta*) have found an inverse correlation between nursing rates and the probability of maternal conception [96,97]. However, the temporal patterning of nursing and maternal energetics are not mutually exclusive mechanisms by which the resumption of cycling occurs. Work on humans shows that the duration of lactational amenorrhoea is also influenced by maternal energy balance, as mothers with relatively higher energy supplies resume cycling sooner [98,99].

Our results do not clearly show that alloparenting benefits mothers energetically. Still, the finding that it decreases lactation effort and accelerates weaning is in line with Ross & MacLarnon's [10] finding that among primates, generally, the primary benefit of alloparenting is to allow mothers to raise infants to weaning age rapidly, reduce their inter-birth intervals, and thereby increase reproductive rates, even if this is at the cost of infant growth and development (but see [9] arguing that alloparenting and infant growth rates are positively correlated across primate species). A similar within-species benefit of alloparenting has been shown in woodland voles (*Microtus pinetorum*) [100] and African wild dog (*Lycaon pictus*) [101], in which alloparents do not increase infant growth or survival rates but decrease inter-birth intervals and thereby improve reproductive rates.

If mothers do not compensate for accelerated weaning through improved nutrient transfer to their offspring, infants may experience restricted growth and become nutritionally independent at lower weights and smaller sizes [14,33,58]. This could increase post-weaning mortality risks for offspring, and alternate explanations for the occurrence of alloparenting would be required. Whether such risks exist in chimpanzees is unknown, but juveniles stay with their mothers for several years after weaning and thus have opportunities for compensatory growth during this time, especially because chimpanzee females may allow juvenile offspring to feed in the same food patches and even share difficult-to–access foods with them (e.g. fruits of *Treculia africana* and *Monodora myristica*, which are two of the foods eaten most often at Ngogo [102]) ([69,103,104], I. Bădescu 2013–2014, unpublished data). We have also observed older siblings and adult males share difficult-to-access foods with infants through tolerated food thefts (I. Bădescu 2013–2014, unpublished data), and alloparents could thus improve infant daily weight gain and growth in a way resembling species where alloparents provision infants (e.g. banded mongoose, *Mu. mungo* [25]; meerkats, *S. suricatta* [24]).

We found that younger infants were handled more than older infants, which is similar to most species [33,76,79,80,105]. Infants of multiparous mothers received more alloparenting than infants of primiparous mothers. This may mean that the correlations we found between infant handling and maternal lactation effort reflect a tendency for experienced mothers to be more permissive of alloparenting. Concurrently, multiparous females might be better able to quicken the weaning process independently of the handling that their infants receive. Superior lactation performance has been reported for multiparous compared with primiparous mothers in Japanese macaques (*Macaca fuscata*) [86]. Given that multiparous females have gone through the lactation process one or more times with previous offspring, they may be able to produce more nutrient-dense milk than primiparous mothers, as in rhesus macaques (*M. mulatta*) [39,43]. More nutrient-dense milk could mean that infants of multiparous mothers can obtain the energy required to grow and develop to weaning age in a shorter amount of time. Alternatively, the parity effect might have occurred because older juvenile siblings were the most frequent alloparents. This explanation is in line with accounts from other chimpanzee populations and with our own reports at the site that when alloparenting occurs, it is often by older siblings [67,68,71,106].

That alloparenting in chimpanzees presents possible fitness benefits to mothers at Ngogo raises questions as to why relatively few data on chimpanzee alloparenting have been published. In some mammals, alloparenting is expressed flexibly and varies from common where it provides fitness benefits, to rare in situations with no benefits (e.g. sperm whales, *Ph. macrocephalus* [4], European badgers, *Me. meles* [29]). Alloparenting in chimpanzees may be most common when certain ecological or social conditions are met and when its benefits are most likely to outweigh the costs [107]. Maternal kin relationships may provide social reason for alloparenting at Ngogo. In mammals where females typically live and reproduce in their natal groups, alloparenting is often biased towards maternal kin (e.g. wedge-capped capuchin, *Cebus olivaceus* [108]; vervets, *Ch. pygerythrus* [11]; ursine colobus, *Colobus vellerosus* [76]; sperm whales, *Ph. macrocephalus* [4]; macaques, *Macaca* spp. [109]; prairie voles, *Mi. ochrogaster* [22]; banded mongoose, *Mu. mungo* [25]; yellow baboons, *P. cynocephalus* [80]; meerkats, *S. suricatta* [3,24]). High mean relatedness between chimpanzee females at Ngogo [37] may facilitate alloparenting. Indeed, preliminary analyses indicate that maternal kin dyads showed more natal attraction and infant handling than non-kin and unknown-kin dyads [106]. However, not all females who have remained at Ngogo as adults continue to associate with their mothers at high rates [110], and how much alloparenting is directed towards younger siblings remains an open question.

The relatively high abundance of food and relatively low variance in fruit availability at Ngogo [93,111,112] means that feeding competition is lower than at other chimpanzee study sites. One consequence could be that mothers have less need to protect their infants. Females at Ngogo frequently travel with preferred female partners, which further reduces the potential for feeding competition [35,36]. Relatively low feeding competition combined with relatively high maternal kin presence at Ngogo could minimize female reproductive competition and allow mothers to be more permissive of alloparenting than at other sites because it is less likely that their infants will receive aggression from conspecifics [8,107]. Low feeding competition might also enable mothers to meet nutritional requirements even when they carry their infants while they are feeding. If so, this would reduce their potential benefits from allowing others to alloparent while they feed. Such an effect night help to explain why we did not find a significant positive correlation between infant handling and maternal feeding time spent feeding.

Fission–fusion dynamics in chimpanzees allow females flexibility with regard to how gregarious they are. Female gregariousness at Ngogo is higher than in other eastern chimpanzee populations, but also varies considerably among individuals [35,75]. The amount of alloparenting that individual infants receive may vary in association with variation in female gregariousness, and mothers may have considerable latitude to associate with individuals who will provide care for their infants. Variation in female gregariousness might lead to differences in receipt of alloparenting, but this would not affect the central finding that mothers who allow more frequent alloparenting exhibited reduced lactation effort. Instead, it would simply raise the question of why not all females took (or could take) advantage of the possible benefits of alloparental care. Also, female gregariousness in chimpanzees depends heavily on the availability of fruit, the main component of chimpanzee diets; neither fruit availability nor the size of parties that include adult females shows consistent seasonal variation at Ngogo, presumably because fruit availability is generally high and stable and periods when fruit is scarce are relatively infrequent [35,113]. Thus, access to alloparents other than older dependent offspring may typically be high, although maternal permissiveness might decrease during times of relative fruit scarcity.

### 4.1. Conclusions and future directions

Data gained from a novel method of stable isotope analysis and from direct behavioural observations showed that the speed with which females weaned their infants varied inversely with the amount of alloparental care their infants received. This relationship could have held because alloparenting increased intervals between nursing bouts, thereby lessening the inhibitory effect of prolactin on the resumption of cycling; because alloparenting allowed mothers to increase their foraging efficiency and thereby improve energy and nutrient transfer to infants and/or hasten their own return to positive energy balance; or both. To reveal whether mothers experience energetic net gains from alloparenting through reduced effort in carrying infants and/or increased feeding efficiency and to assess the effects of alloparenting on infant growth, future studies could measure body size, muscle mass, and energetic differences between individuals by non-invasively using c-peptide and creatinine excreted in urine [114]. Finally, the extent to which alloparenting and maternal permissiveness vary across individuals as functions of parity, availability of maternal kin and current food availability deserve further investigation.

Our findings contribute to the literature showing that alloparenting is a flexible component of female reproductive strategies in some mammals [4,29,101,107]. Our cross-sectional sample shows that alloparenting could be adaptive because it accelerates weaning and thereby increases fertility, but whether this enhances lifetime reproductive success for females remains to be investigated.

## Data accessibility

Data on mean behavioural rates and mean faecal stable nitrogen isotope differences between mother and infants by infant per age category can be found at the Dryad Digital Repository: http://dx.doi.org/10.5061/dryad.02rg3 [115].
